# Care Coordination as Imagined, Care Coordination as Done: Findings from a Cross-national Mental Health Systems Study

**DOI:** 10.5334/ijic.3978

**Published:** 2018-08-23

**Authors:** Ben Hannigan, Alan Simpson, Michael Coffey, Sally Barlow, Aled Jones

**Affiliations:** 1 School of Healthcare Sciences, Cardiff University, GB; 2 Centre for Mental Health Research, School of Health Sciences, City, University of London, GB; 3 Department of Public Health, Policy, and Social Sciences, Swansea University, GB

**Keywords:** articulation work, care coordination, mental health systems, qualitative research, work as done, work as imagined

## Abstract

**Introduction:**

Care coordination is intended to ensure needs are met and integrated services are provided. Formalised processes for the coordination of mental health care arrived in the UK with the introduction of the care programme approach in the early 1990s. Since then the care coordinator role has become a central one within mental health systems.

**Theory and methods:**

This paper contrasts care coordination as work that is imagined with care coordination as work that is done. This is achieved via a critical review of policy followed by a qualitative analysis of interviews, focusing on day-to-day work, conducted with 28 care coordinators employed in four NHS organisations in England and two in Wales.

**Findings:**

Care coordination is imagined as a vehicle for the provision of collaborative, recovery-focused, care. Those who practise care coordination are concerned with the quality of their relationships with service users and the tailoring of services, but limits exist to collaboration and open discussion. Care coordinators describe doing necessary work connecting people and the system of care. However, this work also brings significant administrative demands, is subject to performance management which distorts its primary purpose, and in a context of scarce resources promotes generic professional roles.

**Conclusion:**

Care coordination must be done. However, it is not consistently being done in the way policymakers imagine, and in the real world of work can be done differently.

## Introduction

Long-term ill-health is disruptive. It challenges personal identity, alters social relations and creates uncertainty [1,2]. Episodic crises are liable to happen as symptoms emerge or recur [3]. Problems in one sphere can lead to problems in another. For example, a chronic health problem becomes a social problem once it limits interactions with others, and becomes an employment and income-related problem as paid work becomes difficult to sustain. Just as the nature of long-term illness is a source of complexity for patients, families and those working in care systems, so too is the way services are organised [4]. In modern health and social care systems with developed divisions of labour the formal responsibilities to respond to an individual’s multiple, interconnected, problems rarely fall to a single profession or agency. Knowledge and skill are dispersed, and staff, patients and families find themselves having to work together across practitioner and organisational boundaries to join care up.

It is in this context that effective care coordination is required. Without this, the risk exists that the individual’s needs will remain unmet and/or disconnected services will be provided. In the UK’s mental health services formalised systems for the coordination of care have existed for many years. In England, over a quarter of a century has passed since the introduction of the care programme approach (CPA) [5], with implementation in other parts of the UK following in due course [6]. The appearance of the CPA reflected international trends towards the adoption of case management for people using specialist mental health services [7]. At its simplest, case management is concerned with the coordination, efficiency and effectiveness of services [8]. Beyond this, the multiplication of different approaches and models in the mental health context has challenged attempts to either precisely define [9] or evaluate [10] it. Notable variants include assertive community treatment (ACT), which originated in the USA in the 1970s becoming characterised by a collective, team-based, commitment to comprehensively meeting mental health need [11]. Strengths-based case management grew from social work practice in the USA and championed the idea that all people have abilities and potential which should be nurtured [12], whilst clinical case management evolved into a service provided by specialists sharing a biopsychosocial understanding of mental illness [13]. Other models include brokerage (in which services are connected by case managers who may themselves provide no personal care) and intensive (in which comprehensive needs are met but without the adoption of the team approach typically found in ACT) [14].

At the same time as models of case management have proliferated, the term itself (if not its underpinning principles) has become contested. Some service users have taken issue with the inference that they are ‘cases’, and need ‘managing’ [15]. In one form or another, however, case management (broadly defined) continues to be endorsed as a key component within modern mental health systems worldwide. Variants are found in Australia [16], New Zealand [17], the Netherlands [18] and elsewhere. In the UK, despite longstanding concerns over the extent to which the CPA has improved quality and user experiences, including continuity of care [19,20], its underlying principles have come to be recommended as the right ones for the organisation of services for all people with long-term conditions, and not just those living with mental health problems [21].

Despite this sustained policy and service organisation interest in institutionalising mental health care coordination as a route to improving integration, continuity and service user experiences little is known about what contemporary care coordinators do in their day-to-day practice, or the system context in which their work is done and which therefore also helps to shape its content [22]. A recent metanarrative review of completed studies into community mental health care planning and coordination has reported on three distinct traditions of research in this area [23], but the absence of a sustained programme of exploratory investigation in this field means that gaps between care coordination as ‘work that is imagined’ and care coordination as ‘work that is done’ may have grown without detection. ‘Work as imagined’ reflects standards, guidance and procedures and is what policymakers, regulators and others believe frontline staff are (or should be) doing, whilst ‘work as done’ is the reality of what staff at the sharp end actually do [24,25,26]. Studies of the overlaps and distinctions between the two can identify gaps between policy and practice, and focus attention on the real-life contexts in which roles are fulfilled, the workarounds and adjustments in which frontline staff engage to get their jobs done and the experiences of staff and service users. Care coordination in mental health systems is an instructive case for analysis informed by ideas of this type, as new policies for it have appeared at accelerated pace adding layers to how it is envisaged it is fulfilled. Given that mental health systems are complex, contested and pressed for resources [27,28] much more needs to be known of the tasks completed, and the accommodations made, when practitioners coordinate care in the real world of work.

Against this background we have two aims for this paper. First, via a critical review of policy for England and Wales we consider how policymakers and others at the ‘blunt end’ [26] imagine care coordination to be. Second, having established an ideal of care coordination as it is envisaged we use qualitative data extracted from a large-scale cross-national study in the field of community mental health care to examine how care coordination is actually done, and the system context which shapes this. We compare and contrast these different versions of care coordination as work, and close with lessons learned.

## Care coordination as imagined: from care administration to recovery-oriented practice

The mental health field remains a challenging one for policymakers. This is partly because of the system’s organisational complexity, but also because of the relative lack of evidence to support change and the extent to which policy for mental health becomes intertwined with policy for other areas [29]. Policy for care coordination has not been contentious in the way that policy for compulsory treatment [30], or for redrawing professional boundaries, has [31]. However, the landscape has become increasingly layered as policymakers’ aspirations for care coordination have grown. Over time the CPA in England, and its contemporary Welsh analogue care and treatment planning, have come to be envisaged as overarching frameworks for the provision of care which is underpinned by commitments to certain values. Principal amongst these is ‘recovery’. This is far removed from the original intentions laid out for the CPA when this was first announced in 1990, in a brief circular issued for the joint attention of English NHS organisations and local authorities [5]. In this initiating document the details of how the CPA might work were left to local-level managers, the text being largely confined to a statement of administrative goals. In the face of competing versions of how case management in mental health services might be done (assertively, intensively or via brokerage, for example), no reference was made to underlying philosophy or values [32]. Stated simply, the CPA was introduced with an emphasis on interprofessional care planning and on keyworking, and was directed (as the full title of this first policy document made clear) at “people with a mental illness referred to the specialist psychiatric services”. The broad guidance provided to local services was that the CPA should ensure a systematic approach to the assessment of the needs of all service users in receipt of specialist community mental health care, and to the provision of care by members of the interprofessional team. The role of the keyworker (as the role of care coordinator was initially termed) was described as maintaining close contact with each service user for whom they had responsibility, and overseeing arrangements for ongoing monitoring and timely review. Keyworkers, the initiation document stated, could be drawn from any of the health and social care practitioner disciplines.

The CPA, its local implementation led largely by health care organisations and applying to all people in receipt of secondary mental health services, appeared shortly after the launch of local authority-led care management which had given councils responsibility for the assessment of social care needs and the commissioning of social care packages for all groups of people using community care services [33]. Considerable confusion reigned as parallel systems for case management therefore came to be introduced [34]. Within four years, in the case of people being discharged from psychiatric hospitals England had seen new guidance specifically reminding mental health professionals of the importance of addressing risk and safety (including the risks of harm to self or others) in their care plans [35], and the addition of supervision registers [36]. These were a bolt-on to locally implemented versions of the CPA designed to make sure that care plans were particularly robust, frequently reviewed and well-coordinated in the case of people judged to be at particular risk [37]. One year later *Building bridges*, a substantial document produced by England’s Department of Health for the purposes of guiding interagency care for people with severe mental health problems, described the CPA’s principles of individualised assessment, care planning, coordination and review as the cornerstone of mental health policy [38]. Within two years of the appearance of this document a new government had been elected with a goal of investing in mental health services. Early New Labour was centralising in its health policy for England [39], creating national standards for mental health care to which all local services were expected to aspire [40]. Guidance on the CPA at the end of the 1990s was therefore directive, introducing for the first time at national level two tiers of care planning and coordination based on assessments of service users’ needs and complexity and dropping the term ‘keyworker’ in favour of ‘care coordinator’ [41]. This policy also attempted to close the gap between the NHS-led CPA and local authority-led care management, stating that the two systems should be fully integrated in the case of people with mental health difficulties. Meanwhile, in Wales the principles of care coordination and the keyworker role had been introduced in the middle of the 1990s [42] but it was not until the next decade that a two-tier CPA was formally introduced [6]. In England, guidance on the modernisation of the CPA recommended a single tiered approach and action to streamline procedures via reductions in bureaucracy [43]. Finally, in Wales the CPA came to be superseded through the introduction of new primary legislation, the Mental Health (Wales) Measure [44,45]. This created, in law, care and treatment planning with an associated national template for the recording of plans and a mandate that all people accepted by secondary mental health services should have an identified care coordinator possessing a suitable professional background. A first code of practice followed in due course [46].

A clear shift in policymaking tone over time can be detected across both countries. Early CPA guidance was concerned with the administration of community care, and was characterised by a progressive tightening of care planning and coordination procedures through increasingly specified national guidance issued in response to concerns over the perceived risks posed by people with mental health difficulties discharged from psychiatric hospitals. England’s refocusing guidance [43] and Wales’ first CPA policy [6], however, marked a change in direction by signalling moves towards a more principles-based approach to the organisation and provision of services. Care coordination came to be described as much more than a technical process for the assessment of needs and the planning, coordination and review of care, transforming instead into a vehicle through which care based on recovery values might be organised and provided.

**Figure 1:**
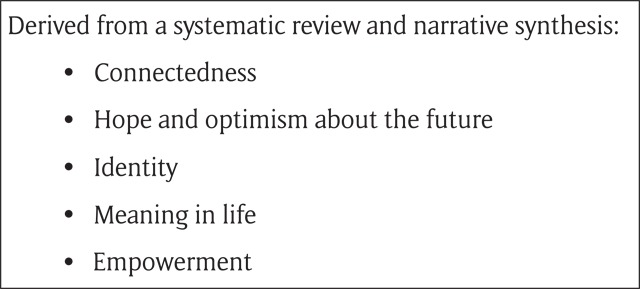
Core concepts underpinning recovery [50].

Originating in the USA [47] ‘recovery’ has come to occupy centre stage in global mental health services. In the UK this has been reflected in a burgeoning literature [48,49], and by attempts to distil the core concepts which might underpin it. A recent summary is given in Figure 1 below.

In recent and current policy guidance care planning and coordination are together envisaged by policymakers as collaborative, tailored, processes through which service users, carers and practitioners might work together in equal partnership in the shared pursuit of recovery goals. Expectations placed on care coordinators are that they place a premium on the quality of their relationships with people using services, assessing needs and planning and coordinating services in ways which reflect shared knowledge of individuals’ hopes, strengths and aspirations. Once the CPA could rightly be criticised for lacking a unifying philosophical base, serving purely as an administrative means of ensuring that people with mental health problems living in the community stayed in touch with services for the purposes of treatment and the management of risk [32]. Policymakers’ more recent attempts to close the gap between the technical planning and coordination of care and the claimed values underpinning service provision make this criticism harder to sustain.

Entirely unknown, however, is the extent to which care coordination as an imagined values-based activity emphasising recovery from mental illness is reflected in the reality of care coordination as it is done. Recent research in this broad area has largely focused on the ways in which care plans are produced and the ways in which people using services might be involved [51], or has examined the experiences of care coordinators [52] in the early years of the CPA. Despite care coordination being seen as key to the organisation of mental health services [53], scant attention has been paid to laying out the day-to-day work that contemporary care coordinators do, the context for this and its consequences.

## The study

A protocol for the project from which data are drawn in the sections following has been published previously [54]. In summary, this was a cross-national comparative case study across six sites (four NHS trusts in England and two local health boards in Wales) which aimed to investigate care planning and coordination in the context of community mental health care. A favourable opinion from what was then the National Research Ethics Service was given (ref: 13/YH/0056A), and usual research governance approvals were secured across each participating NHS organisation. All data were generated in 2013 and 2014, with standardised questionnaires on recovery [55] completed by service users and care coordinators, and on therapeutic relationships [56] and empowerment [57] by service user participants only. Semi-structured interviews were conducted with service providers (n = 67, of whom 28 were interviewed in their capacity as frontline care coordinators), service users (n = 33) and family members/carers (n = 17) (total n = 117). Care plans were reviewed using a standardised template (n = 33). Main findings have previously been reported [58], drawing on descriptive and inferential analyses of quantitative data managed using SPSS [59] and framework analyses [60] of all qualitative data completed with the assistance of the software programme NVivo [61]. A further paper has reported on participants’ competing accounts of ‘risk’ [62].

The analysis presented below draws on the care coordinator interview dataset. In each of the six case study sites access was negotiated to a single interprofessional community mental health team, from which service users (up to a maximum of six per team, stratified by care coordinator) were randomly sampled with invitations to take part in a single, semi-structured, interview. Once a service user had participated in an interview, an invitation was extended to his or her care coordinator to also take part in a single interview. Care was taken not to reveal to this professional the identity of the service user on their caseload who had already been interviewed. The schedule of questions for care coordinators reflected the specific aims of the larger study, which were to identify factors facilitating, and acting as barriers to, personalised, collaborative and recovery-focused care planning and coordination of a kind envisaged in contemporary policy. Questions invited practitioners to talk about their day-to-day activities as planners and coordinators of care, the principles underpinning their work, and factors helping and hindering what they did. Interviews were conducted by members of the core research team and by experienced research support staff directly employed in the NHS. Interviewers had no prior relationships with interviewees, and no direct involvement in service organisation or delivery across any of the six sites in which data were generated.

For this paper a new analysis was completed, again with the help of NVivo. This had a clear focus on understanding how care coordination is actually done from the perspective of those with day-to-day responsibilities for it. Analysis was initiated by one of the authors (BH), who first re-read all transcribed and anonymised interviews in full and coded these data. Codes were both descriptive and theoretical, and were developed in two ways: from an inductive reading of participants’ accounts of their work, and as reflections of the *a priori* analytic interests reflected in this paper [63]. For example, descriptions of carrying out paperwork and other office-based tasks were labelled with the code, ‘care coordination – as administration’, whilst the code ‘care coordination – as articulation work’ was created in advance of its application to the dataset by drawing on theory [64]. As we expand on below, ‘articulation work’ refers broadly to the management of service users’ journeys though systems of care. This initial code list was then reviewed and refined; at this stage (as an example) an early code referring to the work of pulling services together was merged with the code, already introduced above, which captured the idea of care coordination as articulation work. In a third phase of analysis each of the remaining 24 codes was connected, initially by BH, to one of three overarching candidate categories prior to being tabled for discussion and challenge at a data analysis event involving BH, AS, MC and SB. Final refinements to categories, culminating in the thematic presentation of findings below, took place during the production of this written paper.

Information about the 28 care coordinators whose interview data are drawn on is given in Table 1. Most were nurses, and data generated across the totality of the larger project indicated that caseload sizes for care coordinators ranged from 25 (in the site we refer to as ‘Artois’) to 50 or more (in ‘Provence’). Here, and in our data extracts, we use the pseudonyms consistently applied to the six sites across all publications arising from the study (Artois, Burgundy, Champagne, Dauphine, Languedoc and Provence).

**Table 1:** Characteristics of care coordinator participants.

	Care coordinators (n = 28) (%)

Mean age	44 (range 27–62)
Gender^a^	
*Male*	19 (68)
*Female*	7 (25)
Ethnicity^a^	
*White: UK or Irish*	19 (68)
*White: other European*	1 (4)
*Indo-Caribbean*	2 (7)
*Black African*	3 (11)
Profession^a^	
*Mental health nurse*	16 (57)
*Social worker*	6 (21)
*Occupational therapist*	3 (11)
*Psychiatrist*	1 (4)
Education^a^	
*Degree*	7 (25)
*Masters*	6 (21)
*Postgraduate diploma/certificate*	6 (21)
*Diploma/similar*	7 (25)
Time working in mental health services^a^	
*10+ years*	16 (57)
*7–9 years*	6 (21)
*4–6 years*	2 (7)
*1–3 years*	2 (7)
Time working as a care coordinator^a^	
*10+ years*	9 (32)
*7–9 years*	5 (18)
*4–6 years*	6 (21)
*1–3 years*	3 (11)
*<1 year*	3 (11)

^a^ Not all care coordinators provided responses to all biographical questions.

## Care coordination as done

Our findings are presented around three major themes: *Engaging, attending and supporting recovery*, which addresses the relational aspects of work; *Connecting and mobilising*, which refers to the work done by care coordinators to bring service users to the system and the system to service users; and *Accommodating and adjusting*, which points to the ways in which features of the mental health system shape the work that care coordinators do.

### Engaging, attending and supporting recovery

In contrast to versions of case management which emphasise the brokerage of services but not the character of the relationship between professional and service user [14], across all sites care coordinators described relational work as lying at the heart of their work. In some formulations this investment in the quality of relationships was linked directly to the promotion of recovery. In response to a question about what they believe service users get from care coordinators, one participant said how:

They get a full package of care from somebody who does what she says she is going to do, is reliable, gives them a sense of, that their problems are being listened to, they’re being supported in the community, and gives them hope that they’re on the road to recovery and things will get better.

Care coordinator 2, Languedoc (England)

In Wales, a practitioner talking about working with newly referred service users spoke of delaying the administrative aspects of care coordination in favour of trying:

[…] to make the patient feel relaxed as best I can just so it’s, ‘tomorrow you can tell me anything, don’t worry’, so just to make it more open. […] I feel they get more out of it doing that rather than me going in, ‘this is your care and treatment plan, this is what’s going to be happening’ and it’s very, I don’t know, regimental I feel, you just don’t build up a therapeutic relationship or it takes a lot longer to happen if you’re not being quite open and flexible with them.

Care coordinator 3, Burgundy (Wales)

Care coordinators were clear that engaging and attending meant tailoring services, as far as possible, to meet individual needs and placing the person at the heart of the process:

Within the care programme approach, the client’s view of the treatment is very much at the centre of what we’re trying to do and as I’ve said services and care is planned with the person so that the treatment makes sense to both services and the client.

Care coordinator 2, Artois (England)

[…] the important bit for me is the fact this, the patients, it’s sitting down with the patient, it’s their individual care and treatment, it’s theirs, it’s patient focused, it’s tailored to them, that’s what I think’s important, because obviously you can’t treat people generically because everyone’s different.

Care coordinator 3, Burgundy (Wales)

With regards to the wider impact of shifts towards values-based care, some suggested that recovery ideals had exerted an impact on practice and services by encouraging greater clarity around targets and tailored support. Asked to talk about the relationships between care coordination and recovery, a participant said:

I think, it’s definitely become, over the last 18 months or so, become much more focused on setting clear goals with your patient and identifying what you’re going to do with them and delivering that. And then moving the patient through to wherever it is they might need to go to next or discharge, whatever that may be so I think, it’s certainly become more driven in terms of what we want to achieve […]

Care coordinator 3, Languedoc (England)

Although commitments of this type to service user centredness by frontline practitioners reflect prevailing ideas about how care coordination is imagined to be done, participants also indicated that collaboration was contingent on their willingness to share power and on estimations of service users’ fluctuating capabilities. A care coordinator in Dauphine spoke of ‘allowing’ participation, but also of exercising the authority to make decisions in the absence of service user involvement:

It’s about them, it’s their need that we are assessing, this is, we participate, we let them participate as far as they were willing to participate with their care plan […] As far as they can participate we’ll allow that, and then, because planning is continuous, we don’t have to do, and in future if he gets better and can, can contribute more, then he contributes more.

Care coordinator 6, Dauphine (England)

For some care coordinators, conducting open discussions directed at the shared identification of tailored, service user-centred, goals was challenging:

[…] some people do participate, some people don’t want to participate. What they say is that ‘you are the expert, you just do what you want to do’. There are some people who, who know what they want, or what they need, and then try to, to work with the professionals to achieve the goals. Whereas it’s not all the clients that really, really wanted to come there, but in the planning they don’t care, they want to leave that to the professionals.

Care coordinator 4, Champagne (Wales)

An area where commitments to open discussion and transparency were absent was risk and safety. Despite ‘risk’ being described as the single most important domain on which care coordinators focused their energies they did not engage in open discussion with service users about this, a finding previously reported from this study [62] and therefore not pursued further here.

To this point we have shown how care coordinators position the quality of their relationships as a route to the promotion of recovery, in ways which reflect current expectations of how their work should be accomplished. Whilst coordinators engage in relational work focusing on personal goals we have also shown that, at times, they limit the content of their interactions to avoid shared discussions. This is particularly so in the context of recognising and responding to risk. We next turn to care coordinators’ accounts of doing connecting work, of a type which since the first introduction of the CPA in England [5] has consistently been imagined as being central to what they do.

### Connecting and mobilising

A defining characteristic of care coordination as it is imagined is doing work which connects people and the system of care. The need for this arises in the context of both people and the system being complex. The concept of ‘trajectories’ is helpful here, drawing attention to the unfolding of individual experiences of health and illness over time and, critically, the work which is simultaneously done [64]. Trajectories are vulnerable to personal, health-related, contingencies (such as crises and setbacks) but also to contingencies which reflect features of the system. Both have the capacity to knock trajectories off course, and it is because of this that they need to be ‘articulated’ [65].

Care coordinator accounts of articulation work included descriptions of mobilising resources when service users crossed from one part of the system to another. Hospital admission was one:

Let’s say, for instance, if a patient gets admitted to hospital say, for instance, it’s my role then to ensure this is happening, that’s happening, that’s happening, and it’s not necessarily me doing it, I could just care coordinate with the ward and say, ‘[…] when can you do it, or you book it in, you let me know’, it’s just ensuring things are being done […].

Care coordinator 3, Burgundy (Wales)

Responding to the question, ‘What does the service user get from you as a care coordinator?’, one said how:

[…] I hope they would know that I’m the person pulling in the strands and that they’re, of course can chat to their individual clinicians about whatever it is they are working with. But if they felt that there were things that, that weren’t happening and should, or that there were things that they would like and they hadn’t got yet, that I could be the person for them to contact and discuss with them and that I would be the person that would have a chat with them […].

Care coordinator 2, Champagne (Wales)

Asked about how they would describe their role to somebody else, another answered with specific examples of linking the person using services to different parts of the system:

I think as a care coordinator what you basically do is you are the main, you are the manager of provision of care for someone suffering from mental health difficulties. So it’s a person who’s actually organising and overseeing the whole delivery of support to someone and tries to keep a track of the different services and professionals involved while keeping the service user informed of everything what is happening […].

Care coordinator 1, Provence (England)

Pulling the threads together so that service users’ care is joined-up was described as demanding work, with care coordinators needing to drive services in ways that required personal persistence and leadership. One participant said how:

[…] you’ve got to be very strong, you have to be a people person, and you have to have good communication skills to remain professional, to remain professional, and because it’s all about delegation.

Care coordinator 1, Dauphine (England)

Formal review meetings were opportunities for care coordinators, other members of the care team, service users and carers to meet. Care coordinators saw these as important events which needed planning in advance, their scheduling typically organised around the availability of psychiatrists who, in turn, often assumed the role of chair. Reviews happened at varying intervals (typically between three and 12 monthly), took place in a variety of settings, including service users’ homes, and at their best provided a forum:

[…] to sit down, have a look at what’s working and what isn’t working, and what needs changing and that includes obviously medication, with the consultant being there, and if there’s anything else we should be doing that we’re not doing. Often, well sometimes relatives or other agencies come along and they can have their say about what they think we should be doing […].

Care coordinator 2, Languedoc (England)

Thus far we have revealed care coordination as drawing on practitioners’ engagement and relationship skills, and as involving articulation work in mobilising and organising the system to make sure that needs are met, and services are both provided and reviewed. Our final theme attends to the wider context in which this work is done. We show how the practice of care coordination is shaped by features of the wider system, and reveal the implications of this for workloads, administrative responsibilities and professional roles. It is here, in particular, that the greatest gaps emerge between care coordination as work that is imagined and care coordination as work that is done.

### Accommodating and adjusting

Care coordinating meant accommodating and adjusting to the demands of the system. Echoing findings from earlier studies [52], and in the face of repeated policy-driven attempts to reduce the burden [41,43], care coordinators across sites described their work as administratively challenging. Changes in required documentation were one source of additional, unwanted, work. Asked a question about what care and treatment planning meant to them, a participant in Wales responded by saying:

One of the annoying things about it is how the format changes from time to time and that they bring out paperwork and you get all your caseload, you’re told you need to get all your caseload now up to date and you need to use this paperwork, so any previous paperwork isn’t acceptable anymore. You’ve got to use this paperwork now.

Care coordinator 4, Burgundy (Wales)

The setting of performance targets for the completion and updating of care plans meant that care coordinators’ work on care planning could become little more than an administrative chore, as opposed to a collaborative process accomplished in partnership with service users. A care coordinator described how:

[…] it becomes more a paper exercise because they have to meet the targets every month. It’s a reminder, ‘is the care plan done, is that done’? And therefore it becomes like work for the care coordinator to do rather than to be done with the patient and it is for the patients, so it really should be patient centred really.

Care coordinator 2, Dauphine (England)

Care coordinators were also busy, with many having responsibility for the care of large numbers of service users. Some teams were carrying job vacancies, and as one participant put it:

[…] with the best will in the world you can’t do a good job if you have a caseload of 60.

Care coordinator 5, Provence (England)

Opportunities to learn how to care coordinate varied both within and across sites. Some participants talked of having had no training whatsoever. Others emphasised the preparation for care coordination received during initial professional education, or of learning experientially whilst in post. Some described formal in-service training (for example, focusing on the organisation of care planning meetings). A new area for many was learning to use new information technology, competence in which was required in order to complete all the tasks associated with the role. Problems described included the incompatibility of electronic record systems across health and social care organisations, and computerised systems which were cumbersome and not user-friendly. Some, however, had embraced opportunities to use mobile technology in their face-to-face care coordinator work, and notwithstanding problems with the structure of care plans or the patchiness of wireless connectivity were using this collaboratively with some service users:

[…] for other people, when you say, ‘let’s sit and do this care plan together’ it’s great, I’ve got a laptop now, we can sit and type it up, that works. That won’t work for everybody, or and so, so I can do, I can take a laptop to somebody’s house and sit with them and say, ‘I’m really sorry I didn’t design this form, but I don’t like the way it’s written, but let’s ignore that because what it’s doing is a really good thing’. To say ‘this is what your problems are for you, and this is how we’re going to try to help you address them. I know we have to fit it into funny little boxes, but never mind that’, it is a good thing.

Care coordinator 1, Artois (England)

System features exerted an impact on the work of frontline care coordinators in other ways, sometimes with implications for professional roles. Care coordination was work shared by practitioners from a variety of practice backgrounds, often working together as members of integrated teams, and its generic character meant that the allocation of care coordinators was not always done on the basis of service user need. A care coordinator with a professional background in social work, asked to describe their role, said:

[…] I think since our team has become an integrated team, I think we’ve taken on more of a generic mental health worker role. So we don’t necessarily have the situation any longer where we’re like, ‘that person has to have a CPN [community psychiatric nurse] or that person has to have social worker or’, we do match it as best, if there are clear needs, but if it’s more about just monitoring somebody and doing relapse work with somebody then I think it goes to anybody […]

Care coordinator 1, Champagne (Wales)

This same care coordinator later described how they could draw in colleagues with different professional backgrounds and skills to help meet service users’ needs. However, for others, articulating service users’ trajectories was associated with raised expectations that they would individually do all the work necessary (rather than coordinate it), irrespective of workload implications or their preparedness with regard to professional skills and knowledge:

I think sometimes, rather than having more clout, I think there’s an expectation sometimes for you to be doing everything for the individual. That can be a bit of a pain sometimes. I’m just thinking if somebody’s in on the ward, you can be left with all sorts of things because you’re the care coordinator, sort it. Sometimes rather than liaising directly, for example, with the psychiatrist or something, they’ll come through me as the middleman. ‘You’re the care coordinator, sort this out’.

Care coordinator 4, Burgundy (Wales)

Invited to talk about parts of their role that worked both well and not so well, another said how:

I think it’s good to have a main named, definite, person who is, has some responsibility for ensuring that things are done. But it does sometimes mean that you end up doing a lot of unusual things for a person that are, you think, this might have been better done by somebody who knows what they’re doing.

Care coordinator 1, Artois (England)

Whilst some participants spoke positively about the influence of recovery values on their work, others were sceptical of the novelty of recovery ideals and suggested that limited resources meant that care coordination was largely confined to ‘firefighting’:

[…] I think anyone who comes into this line of work, I think we’ve always been recovery-focused. I think the staff are recovery-focused and you do want to make positive changes and you do want to help people avoid major setbacks and major crises and that’s not always achievable, but I think it’s about the picking, helping them pick up the pieces and moving on again. I think that’s always at the forefront of what we think we’d like to be doing. But sometimes it’s not always achievable again because of the resources, it’s just, it is firefighting, it’s managing crisis after crisis, after crisis, so doing that meaningful recovery work takes a back step because you’re managing crisis and risk. So it is difficult.

Care coordinator 1, Champagne (Wales)

For others again, ‘recovery’ was being used as a premise to discharge people from secondary mental health services, sometimes in the face of resistance:

And I think for those patients, the patients that have been under the team for a long time and now as we’re looking at in more recovery focus […] I kind of think they think, ‘right, well, if I can’t keep thinking of aims and outcomes then they’re going to say that I don’t need to be under the team any longer’ because I do find some longstanding patients are really clutching at straws of things […].

Care coordinator 5, Champagne (Wales)

Here, in this final section, we have revealed the system context in which care coordination as work that is done is accomplished. We have shown how care coordination carries administrative burdens, and in a context of limited resources places pressure on practitioners to undertake work which stretches their competence. We have also demonstrated how not all care coordinators embrace ‘recovery’ as a new way of underpinning what they do, and how others see recovery as a smokescreen for the manoeuvring of service users away from mental health care.

## Discussion and conclusion

A recent paper highlights the dangerous consequences for patient safety of designing medical devices for an imagined clinical world, rather than for the actual clinical world which is inhabited by healthcare professionals [66]. Another points to the challenges of implementing clinical guidelines in real-life practice when these have been written for workplaces which do not exist [67], and a third reveals the difficulties and tensions practitioners face when standardised procedures clash with professional judgment [68]. As we have done here, all three articles contrast work that is imagined with work that is done. These observations, and ours developed in this paper, cumulatively show how far this distinction is of more than simply abstract interest. Designing policies, standards or guidance for a world of work which is not real has consequences.

In revealing care coordinators’ workarounds and accommodations our data serve as a reminder of the discretion public services practitioners, at ‘street level’, exercise in getting their jobs done [69]. Professionals make choices, in organisational contexts, and act in ways which both reflect and refute what those at the blunt end imagine them to be doing. Strong, collaborative, relationships between practitioners and service users are highly prized in contemporary mental health services [70] and in the practice of care coordination specifically [71]. In this context, policymakers can be reassured that guidance for care coordination which emphases these relational aspects finds an echo in the words of professionals who, when interviewed, emphasise their capacity to engage and attend in the shared pursuit of recovery. We additionally observe that data in this study were generated during a period of austerity resulting in major reductions in funding for mental health services [72]. Practitioners confirming their ongoing commitments to high-quality relational work focused on recovery exemplify the power frontline staff have to resist, or show recalcitrance to [73], the damaging effects on service provision caused by the loss of resources and short staffing. However, close inspection of our dataset also reveals limits to the effectiveness of this resistance, with some care coordinators drawing attention to the challenges of doing sufficient relational work whilst simultaneously having organisational responsibility for large numbers of service users. Some practitioners also specify other circumstances in which collaborative working with service users cannot be readily accomplished. An example is in the context of discussions around risk, despite this being an area of high priority for care coordinators [62]. Our analysis developed in this paper also suggests that care coordination as imagined values-based work promoting recovery is accepted, and realised, in uneven ways. For some practitioners recovery values provide clarity, and support goal-setting and collaboration. Confirming how recovery ideals are open to abuse [74] our data also show how some care coordinators see these as a continuation of practice-as-usual, or, as has been shown elsewhere [75] and as some activists suggest [76], as a cover for the withdrawal of specialist mental health care and treatment at a time of scarce resources.

The need for care coordination arises in the context of complexity [77], and linking service users, practitioners and organisations has consistently appeared in policymaking versions of how care coordination is imagined to be done. We have given multiple examples of service user trajectories being managed by care coordinators possessing knowledge of local resources and the skills to lead, mobilise and connect. No single route to the development of competence in these areas is revealed in our data, with many practitioners describing ‘on the job’ learning. Care plans and associated paper and electronic documentation, along with face-to-face review meetings, provide opportunities for connecting and mobilising work of this type to be formalised, made visible and recognised. However, these common features of care coordination as work that is done come at a cost. Consistent with previous research into the initial operation of the CPA [52], and despite top-down efforts to simplify the burden [41,43], mental health care coordination still makes major administrative demands on those who do it in a way which standards and guidance do not adequately appreciate. Care plans are expected by those at the blunt end to be consistently co-produced by care coordinators working in partnership with service users, but data displayed in this paper have shown how performance targets and management expectations can pressure care coordinators to prioritise the completion of documentation over collaboration. As has been found elsewhere, strategic targets for care coordination and excessive administrative demands risk obscuring the work which really needs to be done, including engaging in the making and maintaining of sustained, collaborative, relationships [78]. In some critiques, the pressure to get paper (and computer) work done means that mental health care planning and coordination amounts to little more than a ‘three card trick’ which promises much but which, perversely, increases the distance between those who use and those who provide services [79]. In addition, as this and other studies have shown [80], faced with few colleagues to whom onwards referral might be made care coordinators can find themselves under pressure to expand the range of tasks they carry out. In the absence of having other practitioners available to make specific contributions to service user care, in our data care coordinators talk of doing work at the margins of their professional competence. This, in turn, raises a challenge to interprofessional boundaries and preparation for practice, and suggests that care coordination as it is sometimes done drives a blurring of occupational roles. Nothing in formal care coordination policy reveals awareness of these eventualities.

In conclusion, we argue that in an organisational context in which care is provided by many, and in which service users cross multiple interfaces during their journeys within and between organisations, care coordination is work which absolutely has to be done. When done well it connects the person with the system of care surrounding them and is accomplished in interpersonally skilled, collaborative, ways which promote recovery. However, care coordination invariably involves administration, takes place in a context of finite resources, is subject to target-setting and requires knowledgeable and skilled practitioners often in possession of limited opportunities to learn their craft beyond experientially. It can also pull practitioners into areas of work at the margins of their competency. Sometimes it is informed by recovery values, but sometimes care coordinators detect recovery language being used as a means of discharging service users from care. Reducing the gap between care coordination as it is imagined and care coordination as it is done therefore requires greater clarity over the meaning of ‘recovery’, and a rescuing of it from its abuses. To be a truly collaborative, tailored, activity focused on the interpersonally mediated organisation of service users’ journeys through the system, care coordination also needs to be extricated from the culture of performance management which currently envelops it and risks reducing it to a series of box-ticking exercises. To be done as it is envisaged, care coordination means investing in people and administrative resources. In short, care coordination is not consistently being done in the way policymakers imagine, and in the real world of work can be done differently.
